# Synergistic effects of magnesium oxide nanoparticles on tribology and emissions in *Datura stramonium L*. biodiesel-fueled diesel engines

**DOI:** 10.1007/s11356-025-36868-5

**Published:** 2025-08-20

**Authors:** Arunprasad Jayaraman, Michael David Atkins

**Affiliations:** School of Mechanical, Industrial & Aeronautical Engineering, University of the Witwatersrand, Johannesburg, South Africa

**Keywords:** *Datura stramonium L.* methyl ester, Magnesium oxide, Performance, Emission, Tribology

## Abstract

The present study investigates the effects of magnesium oxide (MgO) nanoparticles on the performance and emissions of a single-cylinder diesel engine fueled *by Datura stramonium L*. methyl ester (DSLME) under constant speed and varying load conditions. SEM, TEM, and EDX spectroscopy were used to characterize the MgO nanoparticles. Experimental results showed that compared to DSLME20, the addition of 50 ppm MgO led to a 9.5% reduction in brake-specific fuel consumption and a 6.03% increase in brake thermal efficiency, highlighting the beneficial impact of MgO on thermal efficiency, aside from diesel fuel. Emissions analysis demonstrated significant reductions in carbon monoxide (CO), unburned hydrocarbons (HC), and smoke emissions. At full engine load, the DSLME20 + 50 ppm of MgO blend reduced CO by 7.9%, HC by 10.8%, and smoke by 8.7%, compared to DSLME20. However, nitrogen oxide (NO_x_) emissions increased by 6.3%. Additionally, tribological tests conducted using a four-ball tribometer at 1200 rpm, 75 °C, and a 40-kg load for 1 h revealed that the wear scar diameter for the DSLME20 + 50 ppm of MgO blend decreased by 15.74%, along with a 24.5% reduction in the coefficient of friction compared to the DSLME20. These findings suggest that MgO nanoparticles improve fuel efficiency, reduce emissions, and enhance engine durability and wear resistance in diesel engine applications.

## Introduction

The growing population, the expanding industrial sector, and the uncertainty surrounding fossil fuel prices have intensified the demand for renewable energy sources (Raj et al. 2022). The use of alternative fuels has increased in recent decades to reduce fossil fuel dependency and their environmental impact. Biodiesel is the most promising substitute for renewable resources produced from non-edible and edible sources. It is a viable renewable fuel due to its availability and conversion potential (Raj and Tirkey 2023). Second-generation biofuels offer advantages over first-generation biofuels by avoiding competition with food crops, promoting sustainability, cost efficiency, and reducing land use (Liu et al. 2023; Zhou et al. 2021; Singh et al. 2019; Koria and Thangaraj 2010). In addition, the objectives of biodiesel policy encompass enhancing rural incomes, promoting renewable fuels, supporting domestic oil crops, and reducing climate impact. In agricultural economies, local oil crops are prioritized as feedstocks for biodiesel, with increased support during periods of low vegetable oil prices and high crude oil prices (Byerlee et al. 2016). *Datura stramonium L*. seeds are widely available compared to other crops, as the plant thrives in diverse environmental conditions (Batool et al. 2020). Commonly found in China and India near homes, along roadsides, and in grasslands, *Datura stramonium L*. has been used for centuries for its herbal medicinal properties (Gupta 2020). Biodiesel offers unique benefits but has limitations, including poor cold flow properties, injector clogging, incomplete combustion, and high viscosity, which restrict its widespread application in compression-ignition engines (Sorate and Bhale 2015; Chiu et al. 2004). Recently, researchers have explored the use of nanoparticles in biodiesel blends as oxygen buffers to enhance combustion kinetics, reduce viscosity, and optimize cold flow properties (Zhao et al. 2016; Bhale et al. 2009), thereby making biodiesel a cleaner and more viable alternative to fossil fuels (Modi et al. 2024). Modifying the fuel’s chemical composition by blending it with nanoparticles has also been investigated to improve performance and reduce emissions without requiring engine modifications (Kumbhar et al. 2021; Venu and Appavu 2020). Metal oxides, such as aluminum, copper, cerium, cobalt, titanium, and zinc, have been widely used as nanoparticles to enhance the properties of diesel and biodiesel fuels (Chacko and Jeyaseelan 2020; Anbarsooz 2023; and Mofijur et al. 2024). Due to their elevated surface area relative to volume, magnesium oxide nanoparticles enhance engine performance by optimizing fuel atomization and improving combustion efficiency (Vinayagam et al. 2021; Çılğın 2025; Pachiannan et al. 2025; Dharmaprabhakaran et al. 2020). They also accelerate combustion and reduce emissions, helping to mitigate the elevated NO_x_ levels often observed with biodiesel use (Deviren et al. 2023). Table 1 illustrates the performance and emission characteristics of second-generation biodiesel with various oxide nanoparticles. An important factor in internal combustion engines is lubricity, as fuel flow provides essential lubrication to key components, such as injectors and injection systems, thereby minimizing friction and wear (Rodríguez-Fernández et al. 2019). Biodiesel, recognized as an efficient lubricant, has been widely acknowledged in numerous studies for its contribution to enhanced tribological performance (Shafi et al. 2018; Wadumesthrige et al. 2009; Knothe and Steidley 2005). However, using vegetable oils, including biodiesel, is limited by their advantages and poor thermo-oxidative stability at high temperatures (Fox and Stachowiak 2007). To overcome this limitation, the incorporation of nanoparticles into biodiesel has been extensively studied to reduce the coefficient of friction and enhance wear resistance (Chen et al. 2019). Metal oxides, in particular, have traditionally been used to enhance the heat transfer properties of base fluids. It has recently been discovered that nano-lubricants containing metal oxides can reduce friction and wear (Hossain et al. 2022). Layered nanoparticles offer a potential alternative due to their unique physical properties and large surface area, enhancing lubrication efficiency. Their multi-layered structure enables superior load-bearing capacity, reducing friction and wear in engine components. Their excellent thermal stability ensures consistent performance under high-temperature conditions (Asnida et al. 2018; Bhaumik et al. 2018). Table 2 summarizes the studies on tribological tests using biodiesels with nanoparticles.

**Table 1:**
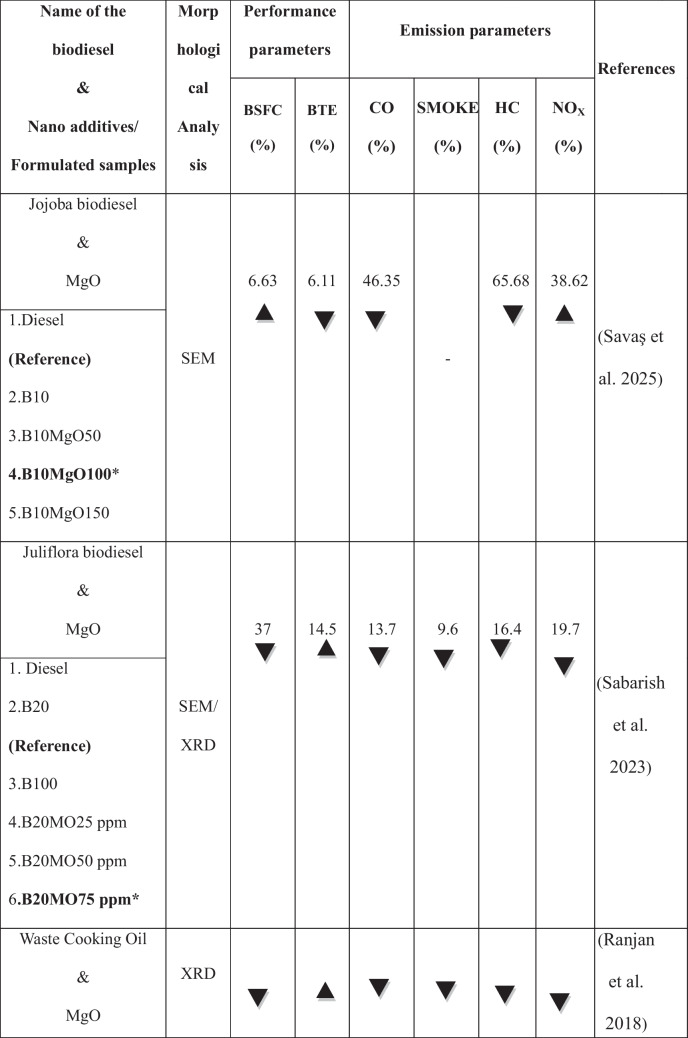

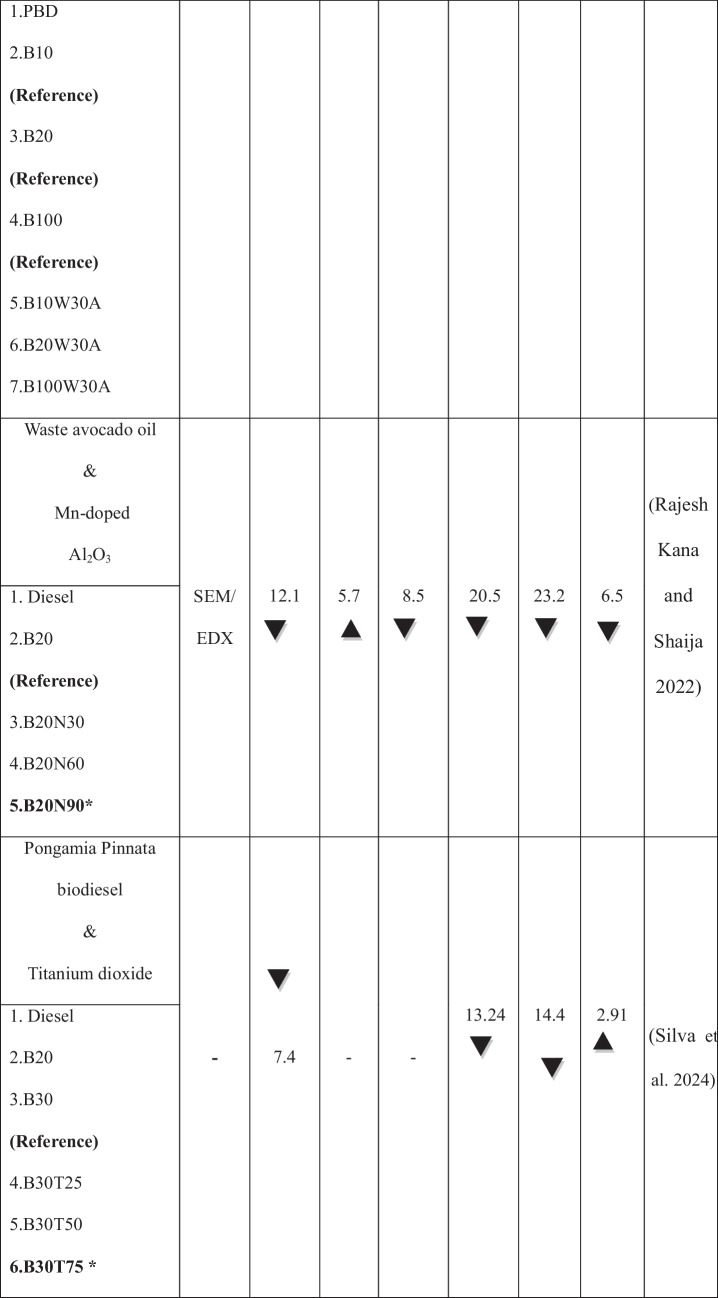

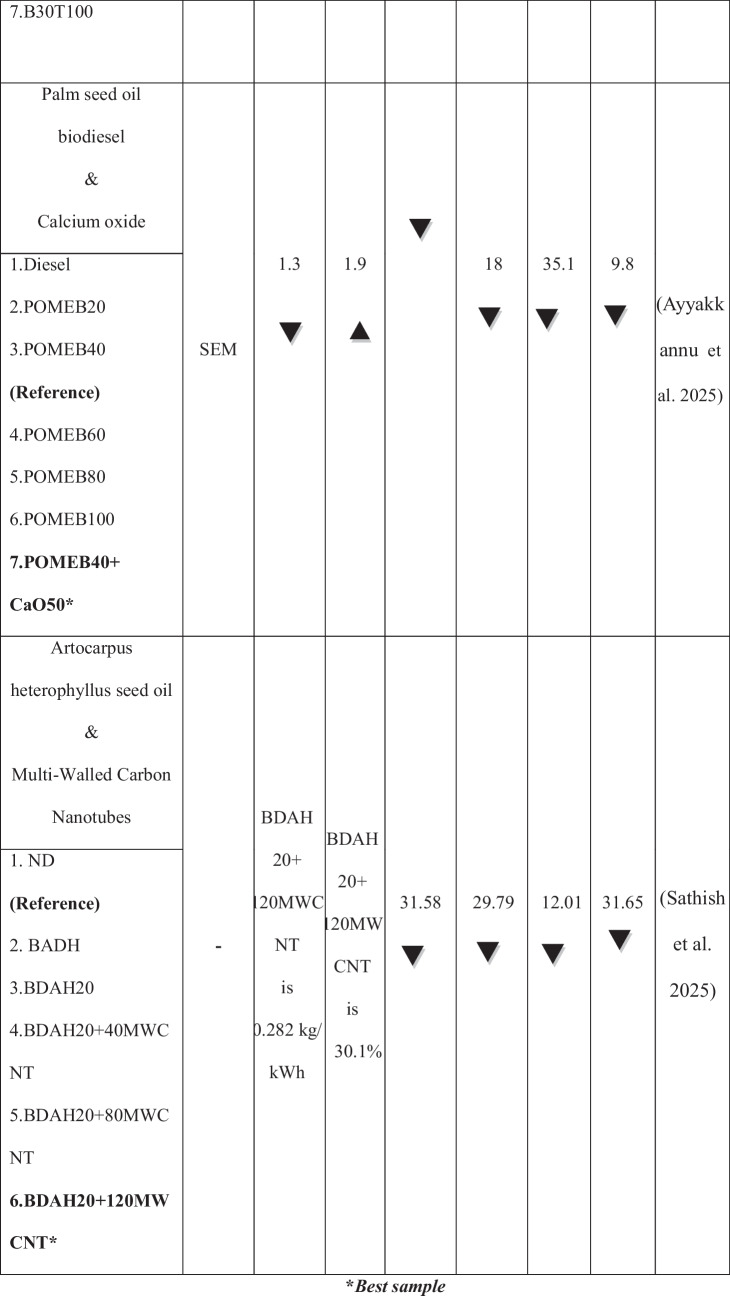
Highlights the performance and emission characteristics of second-generation biodiesel with different nanoparticles

**Table 2:**
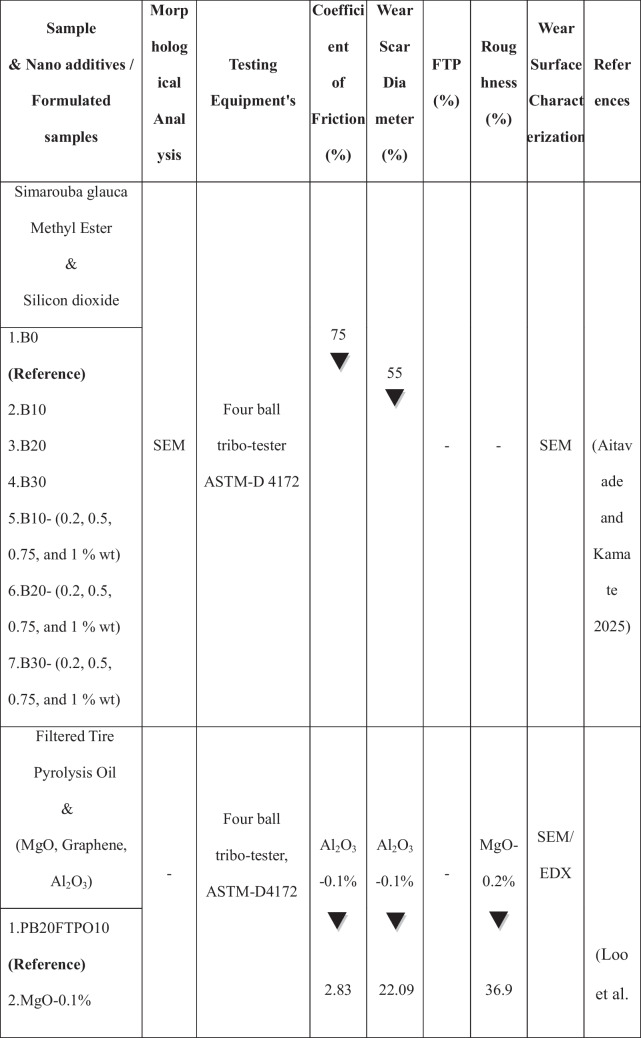

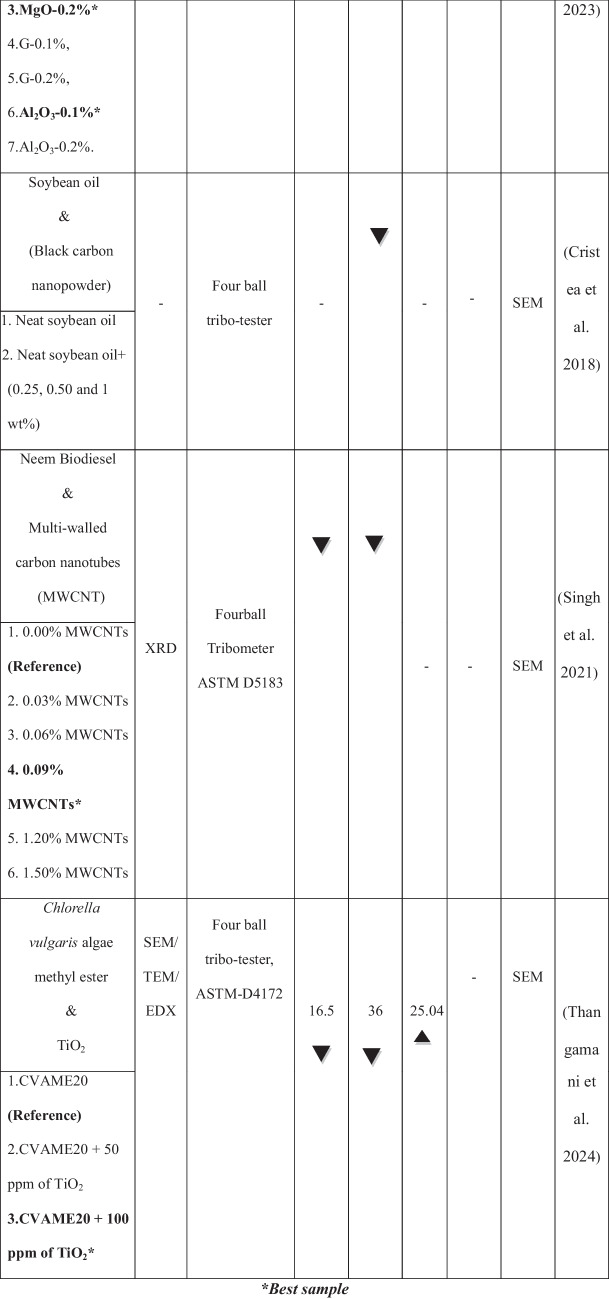
Presents the tribological investigation of several nanoparticles with biodiesels

### Novelty and objectives

The review of existing literature emphasizes that incorporating nanoparticles into biodiesel enhances diesel engine performance, reduces emission parameters and improves tribological properties by minimizing friction between contact surfaces. These findings suggest that nanoparticles play a crucial role in maximizing the efficiency of biodiesel and facilitating cleaner combustion in diesel engines. This research takes a novel approach by using *Datura stramonium L* seed oil as a biofuel source and incorporating MgO nanoparticles as performance-enhancing additives. While the use of non-edible oils with metal oxide nanoparticles in biofuels has been explored to some extent, the application of MgO nanoparticles in Datura stramonium L. methyl ester remains largely unexplored. This study examines the effect of varying MgO nanoparticle concentrations on key diesel engine metrics, including brake thermal efficiency, fuel consumption, and exhaust emissions, such as smoke, CO, NO_x_, and HC. By identifying the optimal concentration of MgO nanoparticles, the research aims to enhance combustion efficiency while minimizing harmful emissions. Additionally, this study investigates the tribological performance of *Datura stramonium L*. biodiesel blended with MgO nanoparticles using a four-ball tribometer. It aims to analyze the effects of MgO concentrations on wear scar diameter and coefficient of friction. This novel approach enhances anti-wear and friction-reducing properties in sustainable lubricants.

## Materials and methods

### Oil extraction and transesterification process

*Datura stramonium L*. seeds were obtained from a local market in Tamil Nadu, India, and dried in an oven at 120 °C for 30 min. The primary feedstock for biodiesel production was obtained after extracting the oil using an oil expeller machine. The crude seed oil of Datura was placed in a container, followed by the preparation of a sodium methoxide solution by dissolving 14 g of sodium hydroxide in 50 mL of methanol. This solution was mixed thoroughly before being added to the oil, which agitated well. The mixture was maintained at 55–65 °C and stirred continuously for 1 h using an agitator. After mixing, the solution was poured into a settling container and allowed to stand for 9–10 h. During this time, glycerin separates and settles at the bottom, while the biodiesel (methyl ester) rises to the top. The glycerin layer was then carefully removed from the biodiesel. The biodiesel was finally collected in a separate container after being rinsed with water to remove any remaining NaOH.

### MgO nanoparticle

MgO nanoparticles were purchased from Sigma-Aldrich Chemicals Private Limited, Bangalore. MgO nanoparticles are recognized for their exceptional thermal stability and high specific surface area, which enhances their effectiveness as combustion catalysts in fuel blends. Table 3 summarizes both chemical and physical properties of MgO.

**Table 3:** The chemical and physical characteristics of MgO

Molecular weight (g/mol)	40.304
Density (g/cm^3^)	3.6
External appearance	White color powder form
Nanoparticle size (nm)	Less than 100 nm
Melting Point (°C)	2852
Band gap eV	7.8

### Preparation of test samples

**Fig. 1:**
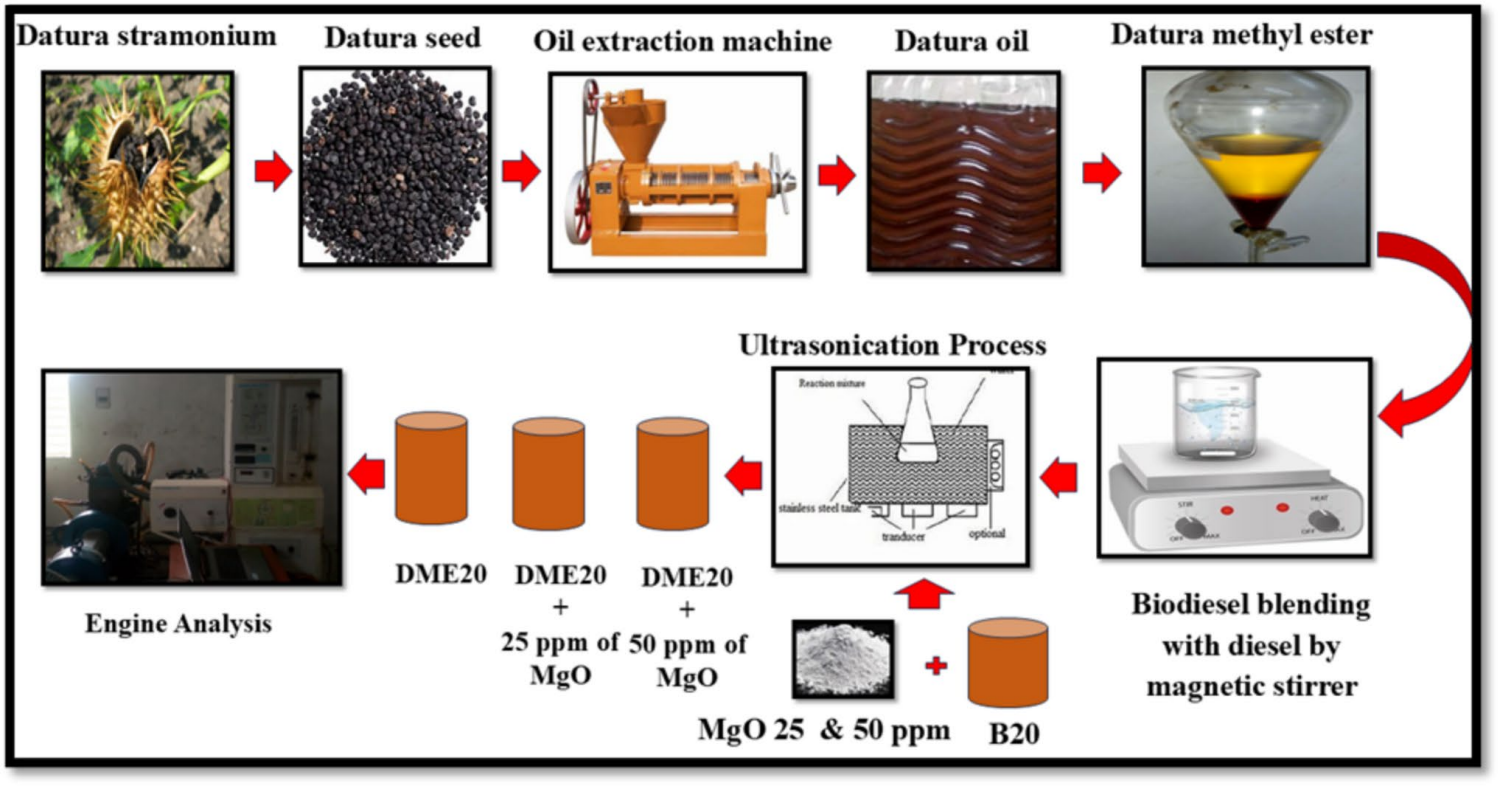
The general procedure for sample preparation and testing utilizing an experimental method

Figure 1 illustrates the overall process of preparing and testing samples using an experimental approach. The experimental fuels were formulated using diesel as the base fuel. Based on existing research, a 20% blend has demonstrated superior performance compared to other ratios (Muni Raja et al. 2018). The present study blended 20% raw *Datura stramonium L*. methyl ester with 80% diesel using a magnetic stirrer to prepare the base blend designated as DSLME20. This blend was then subjected to an ultrasonication process. Initially, it was agitated in an ultrasonication bath for 60 min to ensure consistent mixing. The nanofluid blends were then ultrasonically processed for 20 min at a frequency of 15–30 Hz. Based on prior research, nanoparticle concentrations of 50 and 100 ppm were selected for this study, as several researchers have suggested that these concentrations enhance biodiesel properties, improve combustion, reduce emissions, and optimize engine performance (Rameshbabu and Senthilkumar 2021; Karthikeyan et al. 2015; Hossain and Hussain 2019; and Ramakrishnan et al. 2019). After being weighed, the MgO nanoparticles were added to the DSLME20 fuel blend. The fuel blends were heated to 60 °C and continuously stirred for 30 min using a magnetic stirrer to eliminate residual water molecules. The ultrasonication procedure was repeated to ensure MgO nanoparticles were evenly dispersed throughout the DSLME20 biodiesel–diesel blend. The prepared fuel samples included diesel, DSLME20, DSLME20 + 25 ppm of MgO, and DSLME20 + 50 ppm of MgO. Table 4 presents the chemical and physical properties of different fuel combinations.

**Table 4:** Chemical and physical characteristics of various fuel combinations

Properties	Diesel	DSLME20	DSLME20 + 25 ppm of MgO	DSLME20 + 50 ppm of MgO
Density, kg/m^3^	831	874	854	843
Viscosity, cSt	2.4	3.43	3.52	3.72
Calorific value, MJ/kg	43.4	40.56	41.32	41.89
Flashpoint, °C	70	89	81	78

### Experimental setup

**Fig. 2:**
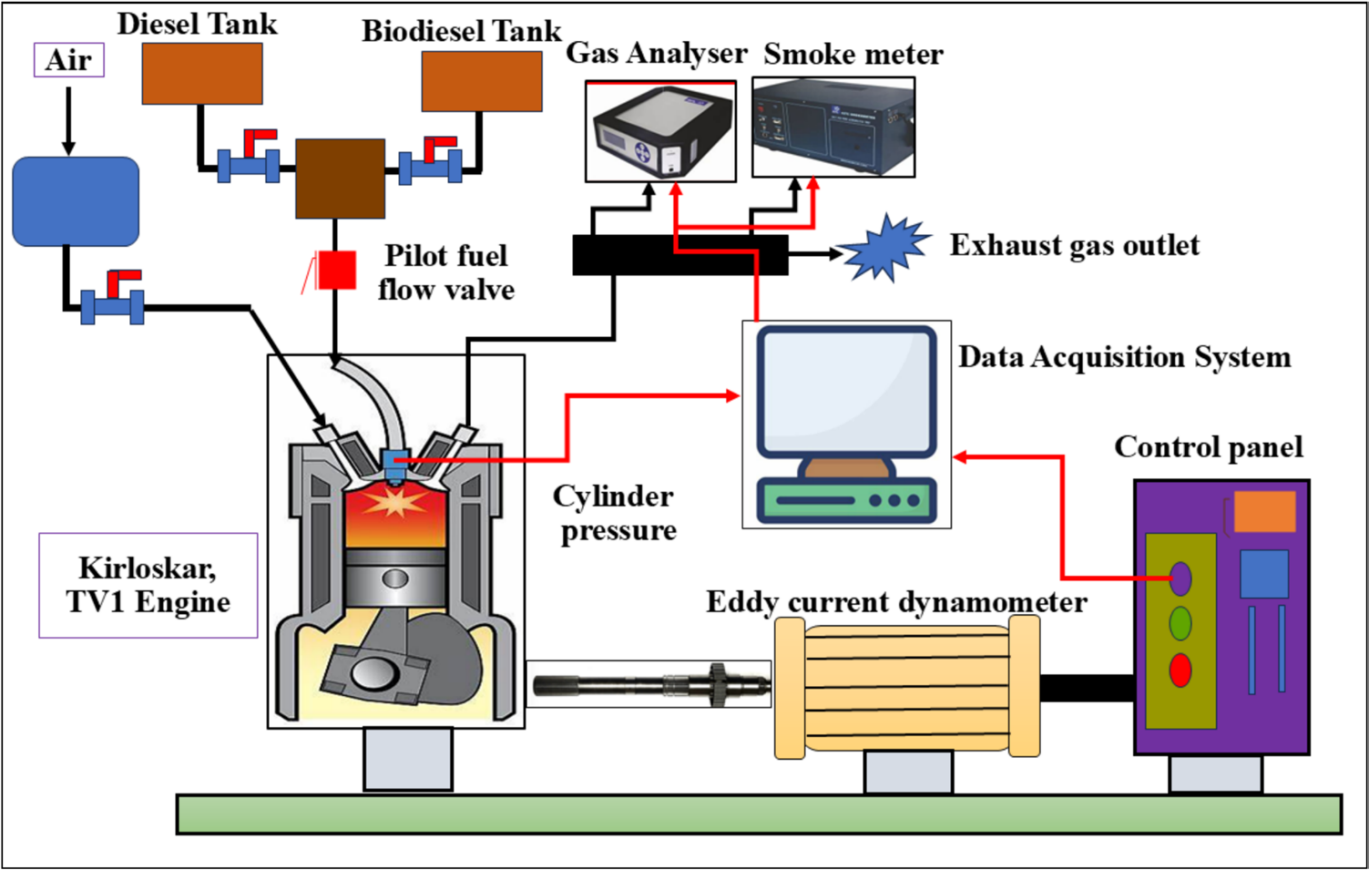
Schematic representation of the test engine configuration

The tests were conducted on a single-cylinder Kirloskar TV1 engine with a rated power of 5.2 kW at 1500 rpm. The fuel injection timing was set to 23° bTDC, with an injection pressure of 210 bar. The engine featured a water jacket that circulated coolant around the cylinder, maintaining a temperature of 80 °C. Cylinder pressure was monitored using a transducer mounted on the cylinder head. A conventional ignition system and an air box were employed to ensure an adequate air supply to the cylinders. The experimental setup consisted of a fuel tank, a fuel control valve, a data acquisition system, and various sensors. A flow meter measured the fuel flow rate and air mixture. The engine operated from zero to full load, with fluctuations regulated by an eddy current dynamometer. Emission parameters were monitored using an AVL 444 DI gas analyzer, and smoke opacity was measured using an AVL 437 smoke meter. Before the actual testing, the engine was ignited and allowed to run for 45 min to stabilize. This stability test was crucial for ensuring accurate data collection and minimizing errors during the recording process. The experimental setup for the engine is shown schematically in Fig. 2. Four different fuels were considered for the experiment on the CI engine, analyzing engine performance and emission characteristics with standard diesel, DSLME20, DSLME20 + 25 ppm MgO, and DSLME20 + 50 ppm MgO.

### Four-ball tribometer

**Fig. 3:**
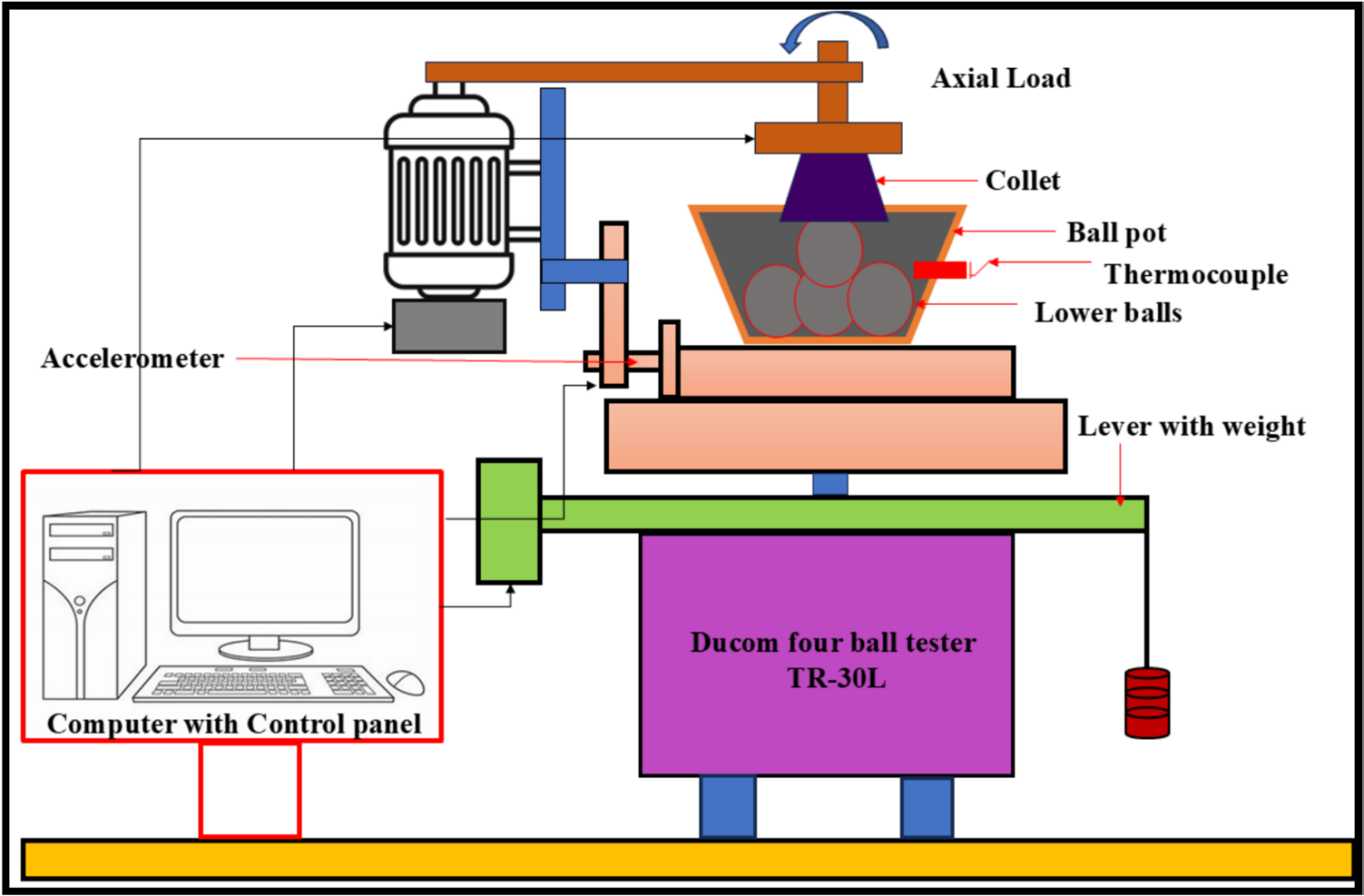
Depicts a schematic illustration of a four-ball tribometer

The four-ball tribometer test evaluates lubricant performance by measuring the coefficient of friction, wear scar diameter, and extreme pressure resistance under controlled conditions. The tribological test in this investigation was conducted using a TR-30L-IAS four-ball tribometer. This apparatus has four balls: the spinning of the collet mechanism propels one steel ball. The remaining three fixed balls are positioned in the oil cup. The steel ball was crafted from chromium-infused steel. Before testing, toluene was used to clean the steel balls and oil cups thoroughly. The test balls were submerged in the oil cup and covered with 10 ml of the test fuel. An experiment was conducted according to ASTM D4172, using a shaft speed of 1200 rpm, a temperature of 75 °C, a load of 40 kg, and a test duration of 3600 s. A data acquisition system was included with the four-ball tribometer to record the frictional torque. An image acquisition apparatus was used to assess the wear scar on the stationary balls. Additionally, an SEM device was employed to examine the eroded surfaces of the spheres. A schematic diagram of the four-ball tribometer is shown in Fig. 3.

#### Coefficient of friction

The frictional torque was measured throughout the investigation using a load cell. Initially, the frictional torque of both test lubricants increased rapidly. The values stabilized after 5 to 10 min, indicating a steady-state condition. A frictional torque analysis was conducted using the appropriate equations to determine the average frictional torque and the coefficient of friction.

$$\upmu =\frac{T\times \surd 6}{r\times 3W}$$

where.

The frictional torque is represented by *T* (kg·mm), the applied load is indicated by *W* (kg), and the distance *r* (measured at 3.67 mm) between the spheres’ contact surface and rotating axis is perpendicular.

#### Wear scar diameter

The wear scar diameter of the three test spheres was measured in accordance with ASTM D4172 to assess the lubricant’s efficacy. Measurements of the initial wear scar diameter were made with an optical microscope. Additional analysis of the wear scar thickness was carried out using micrographs obtained from both optical and scanning electron microscopes. This method was applied to all three bottom balls to accurately determine their wear scar diameters.

## Results and discussions

This chapter examines the impact of blending DSLME20 with varying concentrations of MgO nanoparticles on engine performance and emissions. The study evaluates key performance metrics and emissions under various engine load conditions.

### Characterization of MgO nanoparticle

**Fig. 4:**
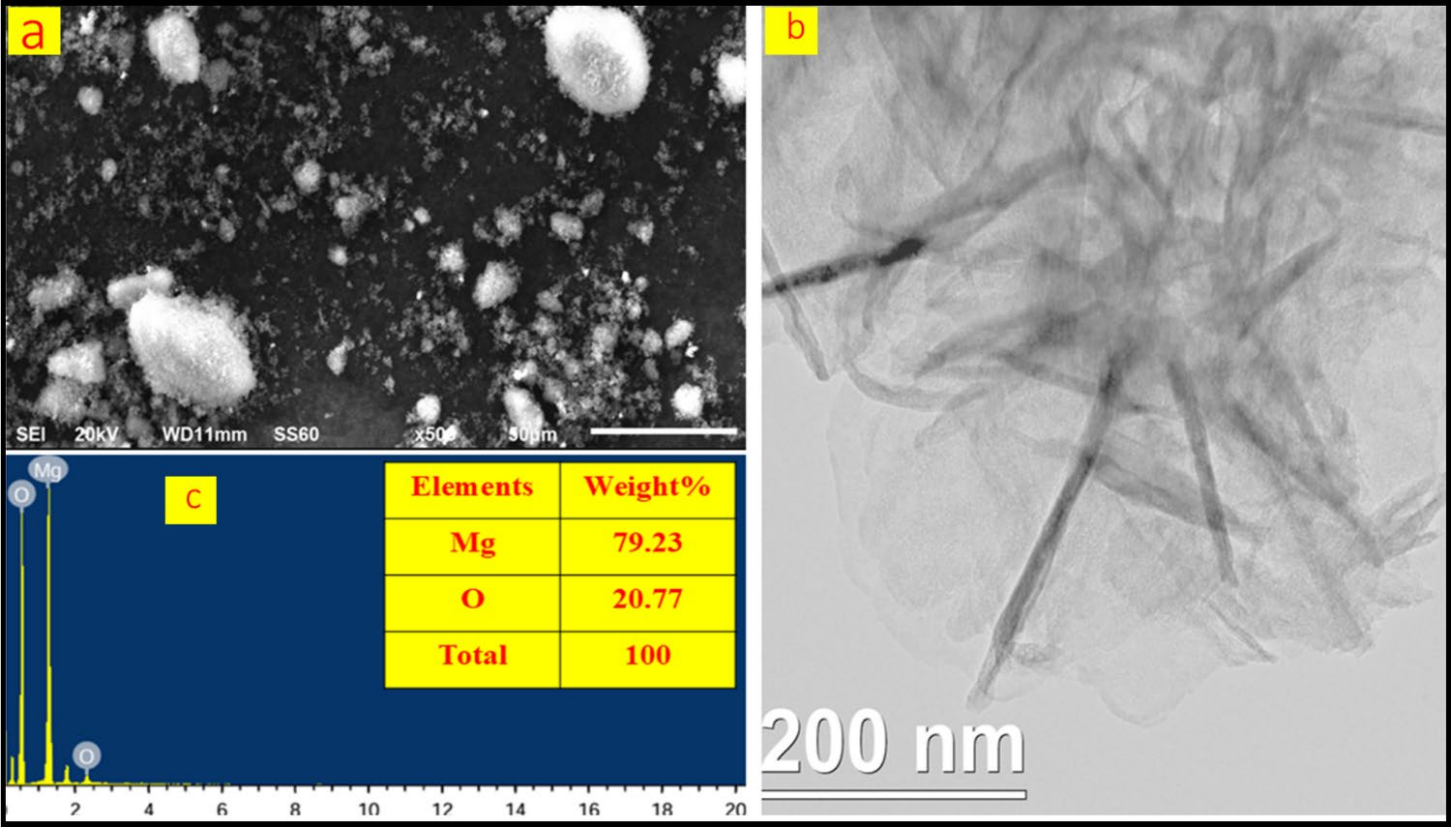
**a**) SEM, **b**) TEM, and **c**) EDX analysis of MgO

The characterization of MgO nanoparticles was investigated using SEM, TEM, and EDX. SEM analysis was performed using the VEGA-3 TESCAN instrument, while TEM analysis utilized the JEM-3010 electron microscope, which features analytical resolution. EDX analysis was conducted using the INCA Energy-250-micron system. This technique enables high-resolution imaging and precise morphological analysis, facilitating the evaluation of nanoparticle properties, microstructures, and surface characteristics. The SEM micrograph (Fig. 4a) depicts the surface morphology of MgO particles at 50 × magnification, revealing white, rounded formations corresponding to aggregated MgO particles with varying shapes and sizes. The TEM analysis in Fig. 4b confirms that the particles are hexagonal, with sizes smaller than 100 nm. Moreover, Fig. 4c highlights that EDX analysis confirms the presence of magnesium in the MgO nanoparticles.

### Brake-specific fuel consumption

**Fig. 5:**
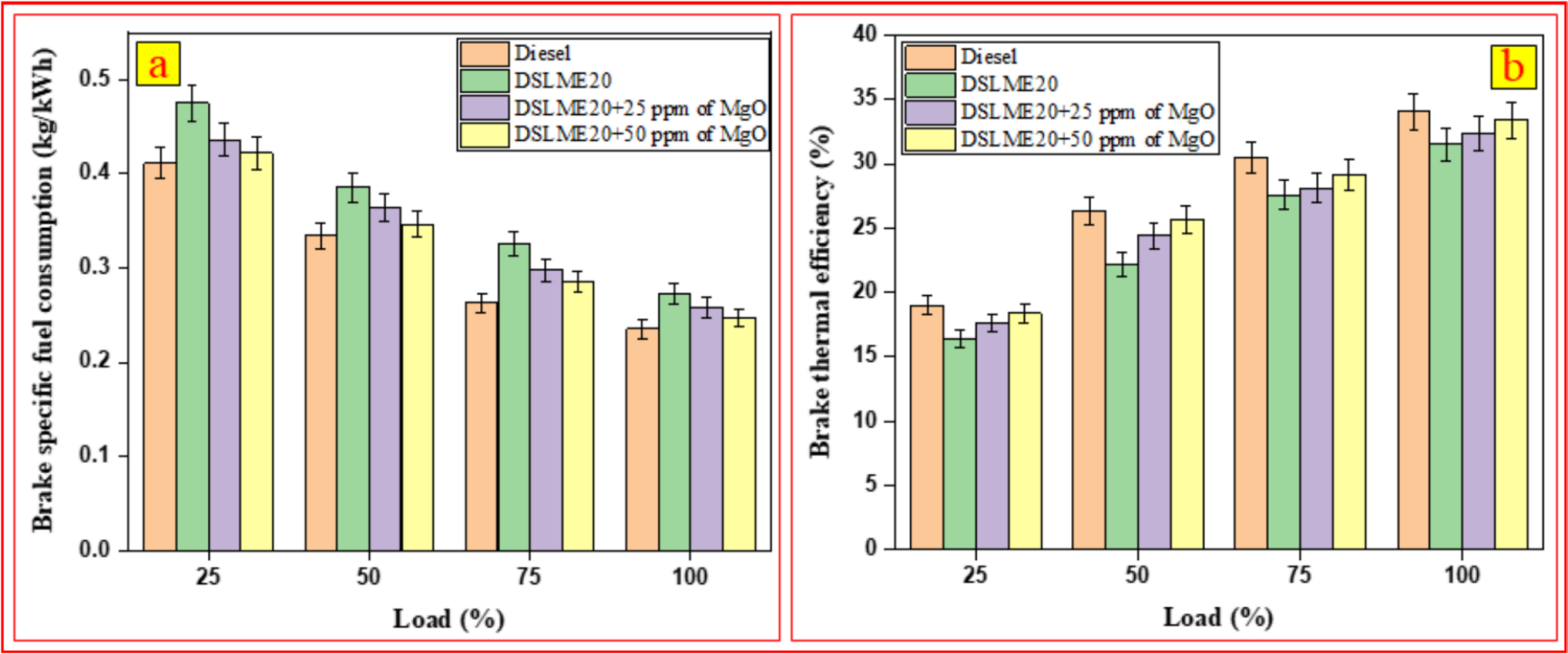
The variation of **a**) BSFC and **b**) BTE in response to load changes

Figure 5 a demonstrates brake-specific fuel consumption of diverse fuel blends under varying load conditions. The findings revealed that BSFC declined when the load increased. Under peak load conditions, the BSFC values for diesel, DSLME20, DSLME20 + 25 ppm of MgO, and DSLME20 + 50 ppm of MgO were determined to be 0.235, 0.273, 0.258, and 0.247 kg/kWh, respectively. The findings confirm that the highest BSFC was recorded for DSLME20. Compared to diesel, DSLME20 exhibited a 16.2% increase in BSFC, while DSLME20 + 25 ppm of MgO and DSLME20 + 50 ppm of MgO showed 9.8% and 5.1% increases, respectively. Incorporating MgO nanoparticles into DSLME20 blends enhanced combustion by facilitating fuel oxidation, improving fuel–air mixing, and increasing flame propagation efficiency, leading to enhanced heat release. Chemically, MgO acted as an oxygen donor, enabling more complete combustion and reducing fuel consumption. This reduction in BSFC indicates that MgO nanoparticles have optimized the combustion efficiency of the fuel blends, thereby enhancing overall engine performance. Compared to DSLME20, BSFC was reduced by 5.5% with DSLME20 + 25 ppm of MgO and 9.5% with DSLME20 + 50 ppm of MgO. Soudagar et al. 2019 observed an 8.3% reduction in BSFC when 40-ppm graphene oxide nanoparticles were added to dairy sludge oil methyl ester.

### Brake thermal efficiency (BTE)

Brake thermal efficiency is influenced by factors such as fuel combustion, oxygen availability, and the preparation of a proper air–fuel mixture. Figure 5 b illustrates the brake thermal efficiency of different fuel blends under varying load conditions. The results show that BTE increases with engine load across all fuel combinations. At maximum engine loads, the rise in temperature inside the cylinder may explain the improved combustion efficiency and the subsequent increase in brake thermal efficiency. Based on the results, the BTE for pure diesel, DSLME20, DSLME20 + 25 ppm of MgO, and DSLME20 + 50 ppm of MgO at 100% engine load were measured at 34.1%, 31.5%, 32.4%, and 33.4%, respectively. Among all tested fuels, traditional diesel recorded the maximum BTE value. Compared to diesel, the BTE for DSLME20 dropped by 7.6%, while DSLME20 + 25 ppm of MgO and DSLME20 + 50 ppm of MgO showed reductions of 4.6% and 2.1%, respectively. In contrast to DSLME20, the brake thermal efficiency increased by 2.86% for DSLME20 + 25 ppm of MgO and by 6.03% for DSLME20 + 50 ppm of MgO, indicating a positive influence of MgO nanoparticles on thermal efficiency. The reason is its efficient fuel–air mixing and better ignition properties. This observation demonstrates that fuel blends with MgO consistently exhibit superior performance compared to the DSLME20 blend under all load conditions except diesel. Furthermore, MgO’s catalytic effect enhances combustion and heat release, increasing BTE by improving fuel-to-energy conversion efficiency. Our findings are consistent with earlier studies. Raju et al. (2018) conducted studies on biodiesel blends incorporating carbon nanotubes and Al_2_O_3_ nanoparticles, which exhibited improved brake thermal efficiency compared to pure diesel. The observed enhancement can be attributed to the elevated heating value of the nanofuel blend. Moreover, the superior oxygenation properties of Al_2_O_3_ facilitated more efficient combustion processes, further enhancing BTE. A steady increase in BTE was also observed as the concentration of nanoparticles increased.

### Carbon monoxide (CO) emission

**Fig. 6:**
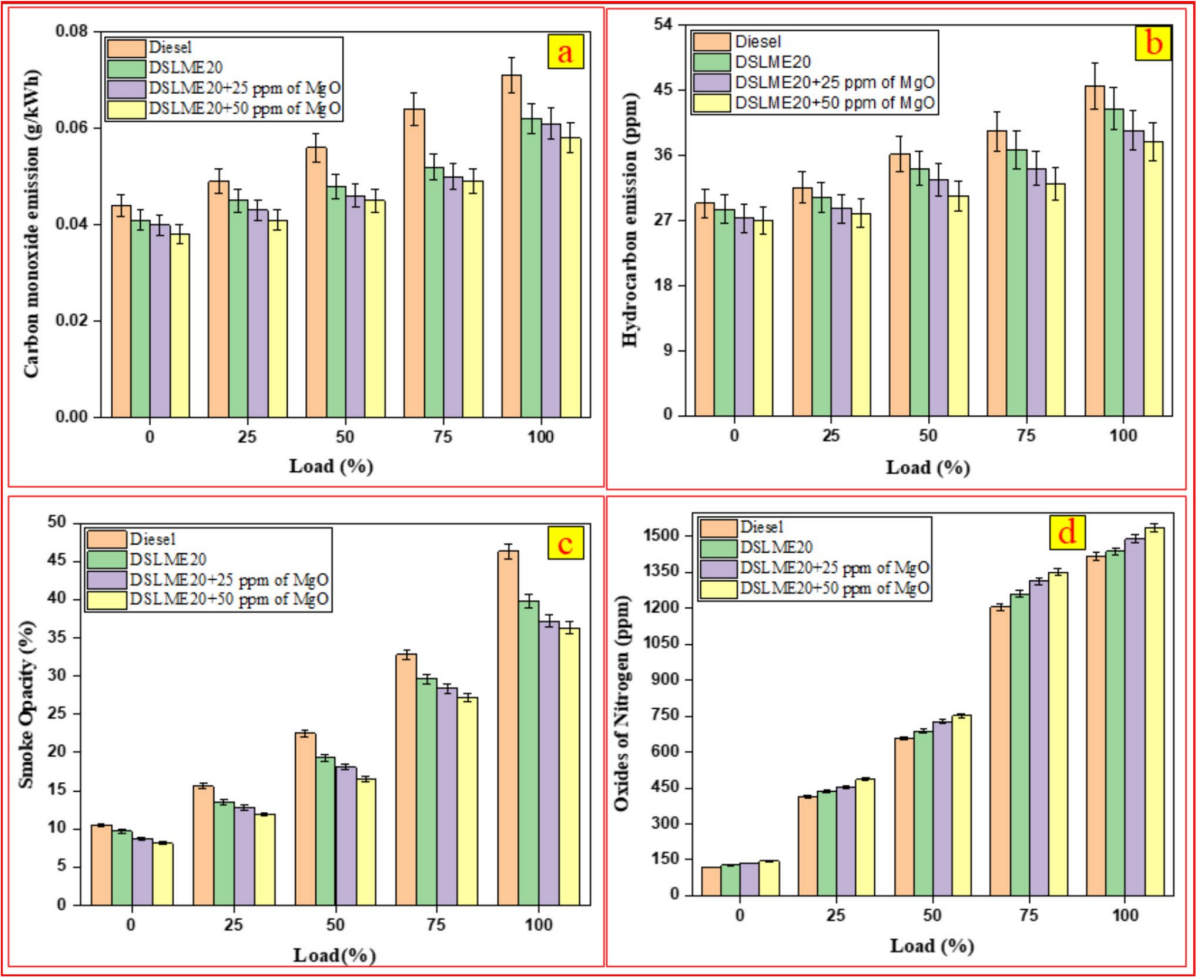
The impact of load on changes in **a** CO, **b** HC, **c** smoke, and** d** NO_x_

Carbon monoxide formation in internal combustion engines is primarily influenced by incomplete combustion resulting from insufficient oxygen or low flame temperatures. Figure 6 (a) presents the variations in CO emissions of the DSLME20 blend at different loads, comparing the effects of nanoparticles with and without their inclusion. A variation in CO emissions was observed among the four fuel blends at maximum load, with diesel and DSLME20 registering the highest emissions of 0.072 and 0.063 g/kWh. At the same time, MgO incorporated fuel blends lowered emissions to 0.06 g/kWh for DSLME20 + 25 ppm of MgO and 0.058 g/kWh for DSLME20 + 50 ppm of MgO. Compared to DSLME20, CO emissions were reduced by 4.7 and 7.9% for DSLME20 + 25 ppm of MgO and DSLME20 + 50 ppm of MgO, respectively. MgO nanoparticles enhance fuel–air mixing and promote more complete combustion by increasing the surface area available for reaction. Incorporating MgO nanoparticles into biodiesel can reduce carbon monoxide emissions by facilitating more thorough and efficient combustion. MgO functions as a catalyst, promoting the oxidation of carbon species and the decomposition of intermediate hydrocarbons. This helps reduce incomplete combustion, a primary source of CO formation, leading to lower CO emissions. Our findings align with previous research. Swaminathan (2023) studied the algae methyl ester incorporated with alumina nanoparticles at various concentrations, ranging from 30 to 120 ppm. CO emissions were reduced by 27.7% in the B20 blend containing 120 ppm ANOP compared to the standard B20.

### Hydrocarbon (HC) emission

Hydrocarbon emissions primarily result from incomplete fuel combustion, generating unburned or partially burned fuel particles. This is often due to inadequate temperatures near the cylinder walls and fuel accumulation in the crevice volumes of the combustion chamber. Additionally, introducing excess air into the chamber can cause lean misfires, reduce flame speed, and generate significant HC emissions. Figure 6 (b) illustrates the variations in HC emissions with different loads for DSLME20 with and without MgO nanoparticles. At maximum load, a notable reduction in HC emissions is observed when MgO nanoparticles are incorporated into the DSLME20 fuel blends. Under peak load conditions, diesel exhibited HC emissions of 45.6 ppm, while DSLME20, DSLME20 + 25 ppm of MgO, and DSLME20 + 50 ppm of MgO recorded 42.5, 39.5, and 37.9 ppm, respectively. The MgO nanoparticles function as oxygen donors, facilitating the oxidation of unburned hydrocarbons during combustion. This improved combustion process results in fewer unburned hydrocarbons, thereby reducing emissions. The DSLME20 + 50 ppm of MgO achieved a maximum reduction in hydrocarbon emissions of 10.8% compared to the base DSLME20 blend. Similar trends have been observed in previous studies, supporting our findings. Ağbulut et al. 2020 reported that adding TiO_2_ nanoparticles to WCO10 fuel reduced hydrocarbon emissions by 70.9% across all load conditions.

### Smoke opacity

Smoke emissions primarily consist of soot, a significant byproduct of combustion, which forms due to the incomplete burning of hydrocarbon fuels. Fuel type, oxygen availability, and engine conditions influence smoke emissions. The engine load has a direct influence on smoke emissions. As the load increases, more fuel is supplied to the engine, resulting in increased soot formation and higher smoke output. Figure 6 (c) presents the trend of smoke emissions at varying load conditions for different fuel types. At maximum load, smoke opacity in the exhaust was recorded as 46.3% for diesel, 39.8% for DSLME20, 37.2% for DSLME20 + 25 ppm of MgO, and 36.3% for DSLME20 + 50 ppm of MgO. The results indicate that higher MgO concentrations can significantly reduce smoke emissions, likely due to enhanced combustion efficiency and lower particulate formation. Compared to DSLME20, smoke opacity was decreased by 6.5% and 8.7% for DSLME20 + 25 ppm of MgO and DSLME20 + 50 ppm of MgO, respectively. MgO nanoparticles inhibit soot formation by enhancing the oxidation of soot precursors and stabilizing combustion, thereby reducing smoke opacity through improved catalytic combustion. Our results are consistent with the findings of previous studies. Perumal and Ilangkumaran 2018 examined the pongamia methyl ester blended with copper oxide nanoparticles. Based on their findings, smoke emissions decreased by 12.8% for the B20CuO100 compared to the base fuel.

### Nitrogen oxide (NO_x_) emission

NO_x_ is among the primary gaseous emissions from internal combustion engines and is known for its high toxicity. The primary component of NO_x_ emissions is nitric oxide, accompanied by smaller quantities of nitrogen dioxide and nitrous oxide. NO_x_ formation depends on three key factors: oxygen availability, combustion temperature, and reaction time. Nitrogen and oxygen react through a chain at elevated engine temperatures, forming NO_x_. Increasing the engine load further raises combustion temperatures, thereby accelerating the production of NO_x_. Figure 6 (d) shows NO_x_ emissions for different fuel blends under varying loads. The results indicate that blending MgO nanoparticles with biodiesel leads to a further increase in NO_x_ levels. At peak engine load, NO_x_ emissions for diesel, DSLME20, DSLME20 + 25 ppm of MgO, and DSLME20 + 50 ppm of MgO were measured as 1417 ppm, 1437 ppm, 1488 ppm, and 1535 ppm, respectively. MgO nanoparticles enhance combustion efficiency by improving air–fuel mixing and flame propagation, as well as acting as oxygen donors. While this reduces unburned hydrocarbons, it also elevates in-cylinder temperatures, which promotes NO_x_ formation. Thus, adding MgO, while beneficial in some ways, can increase NO_x_ emissions. Including MgO in DSLME20 led to a rise in NO_x_ emissions, with increases of 3.4% for DSLME20 + 25 ppm of MgO and 6.3% for DSLME20 + 50 ppm of MgO compared to DSLME20. Sivakumar et al. (2018) conducted a study on a pongamia methyl ester blend (B25) with aluminum oxide nanoparticles at concentrations of 50 and 100 ppm. Compared to the base fuel, NO_x_ emissions were 6.7% and 10.8% higher for B25 containing 50 and 100 ppm Al_2_O_3_ nano additives, respectively.

## Tribology

This chapter explores the tribological performance of DSLME20 fuel enhanced with MgO nanoparticles. The study aims to assess the effects of MgO nanoparticles on wear resistance, coefficient of friction, and overall lubrication effectiveness in DSLME20-based fuel blends.

### Coefficient of friction (COF)

**Fig. 7:**
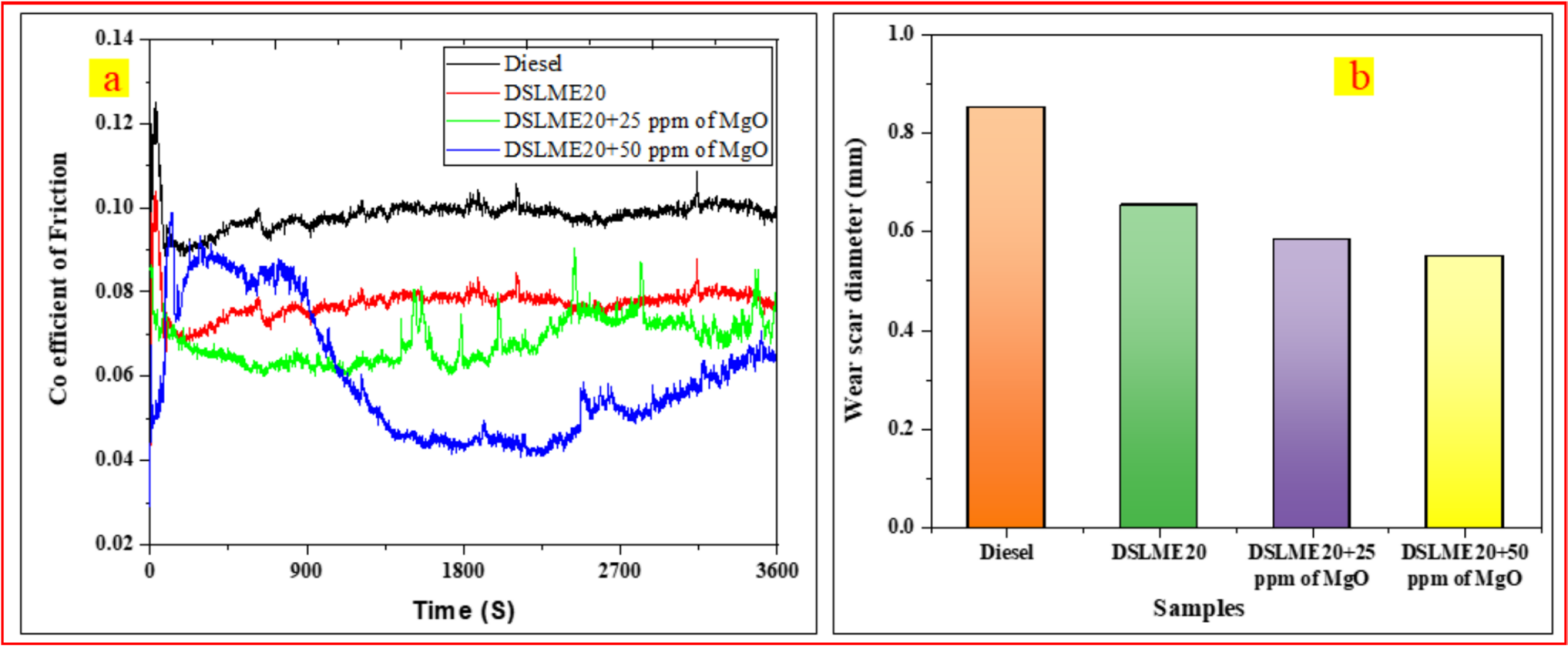
Coefficient of friction Vs. Time **b**) WSD

A considerable increase in friction torque was recorded in all tested samples at the initial stage. A steady-state condition was attained within 5–10 min as the friction torque data stabilized. Figure 7 a illustrates the COF for different fuel blends over time. The COF values for diesel, DSLME20, DSLME20 + 25 ppm of MgO, and DSLME20 + 50 ppm of MgO are 0.1025, 0.08036, 0.07184, and 0.0607, respectively. The coefficient of friction value was significantly higher for diesel than for all tested fuel blends. Compared to DSLME20, the COF of DSLME20 + 25 ppm of MgO and DSLME20 + 50 ppm of MgO is reduced by 10.6% and 24.5%, respectively. This indicates that incorporating MgO nanoparticles into the fuel blend effectively reduces the COF of steel-steel contact pairs compared to DSLME20. The decrease in the COF is due to a structure similar to a lubricating film formed by MgO nanoparticles, which penetrate the surface and integrate into the contact area, creating an additional protective layer. Cortes and Ortega (2019) investigated the addition of CuO and SiO₂ nanoparticles to coconut oil, which reduced the COF by 93.25% and 93.75%, respectively, and the wear volume by 37% and 33%, respectively.

### Wear scar diameter (WSD)

The WSD was measured to assess the wear resistance of the fuel blends. Figure 7 b shows the wear scar diameter for various fuel blends. The WSD for diesel, DSLME20, DSLME20 + 25 ppm of MgO, and DSLME20 + 50 ppm of MgO are 0.852, 0.654, 0.585, and 0.551 mm, respectively. Experimental results reveal that incorporating MgO nanoparticles into biodiesel blends significantly reduces the wear scar diameter compared to other tested samples. MgO nanoparticles in biodiesel blends improve tribofilm formation, creating a protective shield that minimizes wear between metal surfaces (Luo et al. 2014; Zulhanafi et al. 2017). The incorporation of MgO nanoparticles into DSLME20 enhances its wear resistance. Compared to DSLME20, WSD was decreased by 10.5% and 15.74% with the addition of DSLME20 + 25 ppm of MgO and DSLME20 + 50 ppm of MgO, respectively. Baskar et al. (2015) reported that chemically modified rapeseed biodiesel is incorporated with different nanoparticles, including CuO, WS_2_, and TiO_2_, at a concentration of 0.5 wt%. According to the results, the addition of CuO, WS_2_, and TiO_2_ nanoparticles reduced the WSD by 39%, 36%, and 34%, respectively.

### SEM analysis

**Fig. 8:**
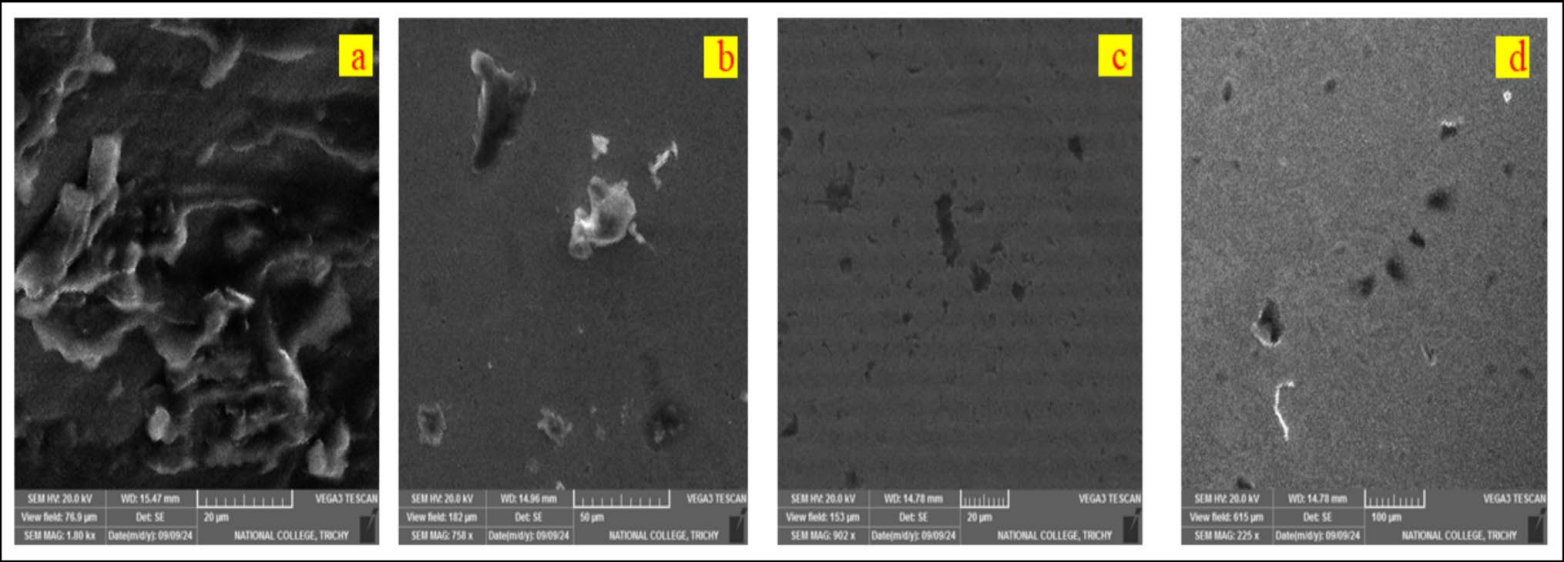
Worn surface characterization of the samples. **a** Diesel. **b **DSLME20, **c**) DSLME20+25 ppm of MgO, and **d**) DSLME20+50 ppm of MgO

SEM offers high-resolution surface morphology and microstructure analysis, enabling detailed examination of wear scars on steel ball samples. Figure 8 provides a detailed visual representation of the worn surfaces. Figure 8 (a) and (b) show SEM images of diesel and DSLME20, which illustrate metal-to-metal contact and abrasive wear mechanisms. Based on the SEM results, Fig. 8(a) and (b) highlight the increased cracks, material removal, scratches, and delamination on the worn ball surfaces. The shadowy appearance is attributed to oxidation (Kharabati et al. 2021). In contrast, Fig. 8(c) and (d) exhibit smoother surface morphologies with fewer cracks and pits compared to those of diesel and DSLME20. The findings of this study revealed that adding MgO nanoparticles to DSLME20 blends enhances tribological properties by forming a protective film between rubbing surfaces, contributing to surface enhancement through a polishing effect. This leads to smoother surface scratches and the elimination of furrows, confirming the superior anti-wear performance of MgO additives. Additionally, the mending effect occurs as MgO nanoparticles are deposited in the valleys of mating surfaces, compensating for worn-out areas and enhancing surface integrity, as demonstrated in the SEM images.

## Conclusion

The experimental study reveals that incorporating MgO nanoparticles into DSLME20 biodiesel blends significantly improves engine performance, emission characteristics, and tribological properties. The key findings are as follows:

- The MgO nanoparticles were characterized using SEM, TEM, and EDX.
- Based on the results, diesel fuel demonstrated the lowest brake-specific fuel consumption among all the tested blends. Compared to DSLME20, BSFC was reduced by 5.5% with DSLME20 + 25 ppm of MgO and 9.5% with DSLME20 + 50 ppm of MgO.
- Compared to DSLME20, the brake thermal efficiency increased by 2.86% for DSLME20 + 25 ppm of MgO and by 6.03% for DSLME20 + 50 ppm of MgO, indicating a positive influence of MgO nanoparticles on thermal efficiency.
- Regarding emissions, the addition of MgO nanoparticles resulted in notable reductions in carbon monoxide, hydrocarbon, and smoke emissions.
- The DSLME20 + 50 ppm of MgO blend at full engine load outperformed DSLME20 by reducing carbon monoxide by 7.9%, unburned hydrocarbons by 10.8%, and smoke emissions by 8.7%.
- However, adding MgO nanoparticles increased nitrogen oxide emissions, with a 6.3% increase observed for the DSLME20 + 50 ppm of MgO blend, reaching 1535 ppm compared to 1437 ppm for the baseline fuel.
- Tribologically, the MgO nanoparticles significantly improved the wear resistance of the fuel blends.
- The WSD for the DSLME20 + 50 ppm of MgO blend decreased by 15.74%, from 0.654 to 0.551 mm, compared to the baseline fuel. Furthermore, the COF was reduced by 24.5% for the DSLME20 + 50 ppm of MgO blend, highlighting the superior lubricating properties provided by the nanoparticles.

In conclusion, integrating MgO nanoparticles into DSLME20 biodiesel blends leads to improved engine performance, reduced emissions, and enhanced tribological properties. These findings suggest that MgO nanoparticles hold promise for optimizing biodiesel-based fuels for use in internal combustion engines**.** Further research is necessary to improve the understanding and optimization of MgO nanoparticles in fuel blends. First, a wider range of MgO concentrations should be explored to determine the optimal level that improves combustion efficiency while reducing nitrogen oxide emissions. Additionally, long-term endurance tests are crucial for assessing the wear and maintenance effects of MgO-enhanced fuels over extended use. Ultimately, evaluating the performance of MgO nanoparticles in various biofuels and alternative fuels would provide valuable insights into their broader applicability, contributing to more sustainable and efficient engine operations.

## Data Availability

All relevant data are available from the authors upon reasonable request.

## Nomenclature

Al_2_O_3_: Aluminum oxide
ANOP: Alumina oxide nanoparticle
ASTM: American Society for Testing and Materials
BTE: Brake thermal efficiency
BSFC: Brake-specific fuel consumption
CO: Carbon monoxide
COF: Coefficient of friction
CVAME: *Chlorella vulgaris* Algae methyl ester
CuO: Copper oxide
DAQ: Data acquisition
DSLME: *Datura Stramonium L.* Methyl ester
DSLME20: 20% Diesel + 80% *Datura Stramonium L.* methyl ester
DSLME20 + 25 ppm of MgO: 20% Diesel + 80% *Datura Stramonium L.* methyl ester + 25 ppm of MgO
DSLME20 + 50 ppm of MgO: 20% Diesel + 80% *Datura Stramonium L.* methyl ester + 50 ppm of MgO
EDX: Energy dispersive X-ray
HC: Hydrocarbons
MgO: Magnesium oxide
min: Minute
ml: Milliliter
min: Minute
MWCNT: Multi-walled carbon nanotubes
NaOH: Sodium hydroxide
ND: Neat diesel
NO_x_: Nitrogen oxides
PBD: Pure biodiesel
rpm: Revolutions per minute
SiO_2_: Silicon dioxide
SFC: Specific fuel consumption
TEM: Transmission electron microscopy
TiO_2_: Titanium dioxide
WCO: Waste cooking oil
WSD: Wear scar diameter
WS_2_: Tungsten disulfide
wt: Weight
